# Osteocalcin and Abdominal Aortic Calcification in Hemodialysis Patients: An Observational Cross-Sectional Study

**DOI:** 10.3389/fendo.2021.620350

**Published:** 2021-03-19

**Authors:** Fengyu Jia, Suxia Wang, Ying Jing, Hanhui Zhao, Peng Rong, Hongbin Zhang, Wenting Lu, Yan Xue, Gang Sun

**Affiliations:** ^1^ Department of Nephrology, The 960th Hospital of the PLA Joint Logistics Support Force, Jinan, China; ^2^ Department of Medical Imaging, Affiliated Hospital of Shandong University of Traditional Chinese Medicine, Jinan, China; ^3^ Department of Medical Imaging, The 960th Hospital of the PLA Joint Logistics Support Force, Jinan, China

**Keywords:** osteocalcin, maintenance hemodialysis, abdominal aortic calcification, vascular calcification, chronic kidney disease-mineral and bone disorder

## Abstract

**Objectives:**

To investigate the serum level of osteocalcin (OC), also known as bone Gla protein, in maintenance hemodialysis (MHD) patients and its correlation with abdominal aortic calcification (AAC).

**Methods:**

From July 2017 to February 2020, we enrolled 108 adult MHD patients. Routine fasting blood laboratory tests were performed before the start of the second hemodialysis in a week. Abdominal aortic calcification score (AACs) was assessed within 1 month. Pearson correlation and Logistic regression were used to analyze the data.

**Results:**

The OC level was 231.56 (25.92,361.33) ng/ml, elevating significantly in this group of MHD patients. It had a positive correlation with serum phosphorus (r = 0.511, P = 0.001), intact parathyroid hormone(iPTH) (r = 0.594, P = 0.0001), fibroblast growth factor 23(FGF23) (r = 0.485, P = 0.003) and a negative correlation with age(r = -0.356, P = 0.039). Based on the AACs, patients were divided into two groups. Serum OC level were higher in patients with AACs≥5 (p=0.032). A multiple logistics regression analysis revealed that age (odds ratio [OR]1.14, P=0.005) and OC(OR=1.10, P=0.008)were risk factors for high AACs(≥5).

**Conclusion:**

The study implicated that OC elevated significantly in this group of MHD patients.OC is positively correlated with phosphorus, iPTH, FGF23, and a negative correlation with age. OC was a risk factor for vascular calcification in this study, but this study did not classify osteocalcin as c-OC and unOC. Whether unOC is associated more directly with vascular calcification requires further study.

## Introduction

Chronic kidney disease–mineral and bone disorder (CKD–MBD) is a common complication in end-stage renal disease(ESRD). CKD–MBD is characterized by abnormal mineral markers, abnormal bone metabolism, and soft tissue calcification, such as vascular calcification (VC) (1). VC is the major cause of cardiovascular disease (CVD) in ESRD, including ischemic cardiac events and subsequent vascular damage. Cardiovascular calcification contributes to approximately 50% of all deaths in hemodialysis patients (2). In contrast, MHD patients without VC always have a better prognosis with limited progression of VC over a longer period of time (3).

Abdominal aortic calcification(AAC) is highly prevalent in the hemodialysis population (4). Kauppila et al. described an AAC grading quantification method by using lateral lumbar radiography in a Framingham study subgroup (5). They showed that this method was predictive of cardiovascular events and mortality (6). Kidney Disease Improving Global Outcomes (KDIGO) in their international clinical guideline for the management of CKD-MBD suggested that lateral lumbar radiography should be used as an alternative to computerized tomography to assess VC (1).

The pathophysiologic mechanisms underlying the process of VC in ESRD have not yet been fully elucidated. VC has been associated with numerous traditional cardiovascular risk factors, including advanced age, hypertension, diabetes, and dyslipidemia, as well as with nontraditional cardiovascular risk factors, such as hyperphosphatemia, hyperparathyroidism, and excessive calcium intake. Today VC in ESRD is considered as an active, complex process, where the first, crucial step toward calcification is the transformation of vascular smooth muscle cells (VSMCs) to osteoblast phenotype, a process similar to bone formation (7).

Osteocalcin (OC), also known as bone Gla protein, a highly specific marker involved in bone formation, is the most abundant non-collagenous peptide found in the mineralized matrix of bone, concomitantly being an intriguing hormone and expanding the endocrine function of the skeleton with far-reaching extra-osseous effects. OC is produced primarily by osteoblasts in bone, it is also produced locally by VSMCs. The detection of OC in circulation raised the question whether it is involved in the process of arterial mineralization. The carboxylation pathway, vitamin K mediated, is pivotal for the transformation of OC from the undercarboxylated form (ucOC) into the fully functional carboxylated form (c-OC). CKD patients often show a subclinical vitamin K deficiency (8). Schurgers et al. evaluated inactive, dephosphorylated, uncarboxylated OC(dp-ucOC) levels in a cohort of 107 patients whose kidney function varied from CKD stages 2–5D. They reported that levels of plasma dp-ucOC increased progressively with CKD worsening (9).

### Aim of the Study

This study aimed to explore the relationship between the serum OC level and other indices of VC and to determine whether serum OC level is the risk factor affecting VC in MHD patients with CKD–MBD.

## Materials and Methods

The study protocol was approved by the ethics committee of 960th Hospital of the PLA Joint Logistics Support Force (protocol number: 20140214). The study conforms to the principles outlined in the Declaration of Helsinki, and written consent was obtained from all patients before the enrollment.

### Study Design and Subjects

**Figure 1:**
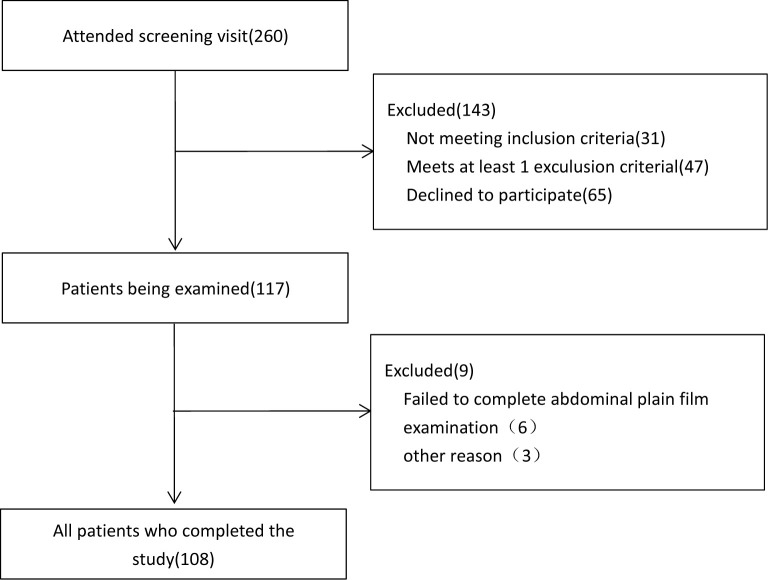
Flow of participants.

We performed an observational cross-sectional study on 108 clinically stable MHD patients, enrolled from July 2017 to February 2020, at the blood purification center of the 960th Hospital of the PLA Joint Logistics Support Force. They were screened continuously from 260 patients in our department. All of the 108 subjects completed the study (**Figure 1**). The study included 66 men and 42 women (age: 24–77 years), and the average age was 55.1 ± 13.6 years. The primary disease was chronic glomerulonephritis in 45 cases (41.7%), IgA nephritis and hypertensive renal damage in 12 cases each (11.1%), diabetes mellitus (DM) (13.9%) in 15 cases, polycystic kidney (5.6%) in 6 cases, and uric acid nephropathy (2.7%) in 3 cases. There were 15 cases (13.9%) with unknown causes.

The inclusion criteria were as follows: (1) age not less than 18 years, (2) dialysis vintage for more than 3 months, (3) patient or legally accepted representative willing to sign Data Release Consent Form. The exclusion criteria were as follows: (1) patients’ life expectancy <6 months. (2) patients with acute kidney injury, active inflammatory diseases, parathyroidectomy, evident malignancies, and abnormal liver function, (3) concomitant diseases that affect calcium status and soft tissue calcifications (sarcoidosis, multiple myeloma, HIV, amyloidosis),(4)patients on anticoagulants such as warfarin.

### Methods

The dialysis regimen for all MHD patients was thrice a week, 4 h per session. The dialysate calcium concentration was 1.5 mmol/L, blood flow was 200–260 mL/min, and dialysate flow was 500 mL/min. The recommended protein intake ranged from 1 to 1.2 g/kg per day. The drugs dose administered for regulating calcium, phosphorus, and intact parathyroid hormone (iPTH) was as per the 2017 KDIGO Chronic Kidney Disease-Mineral and Bone Disorder Guideline Update (10). Clinical and instrumental examinations were performed during the middle of the week, before the HD session. All blood samples were collected before the start of the second hemodialysis in a week in the morning after an overnight fast. Intact PTH were measured by electrochemiluminescence on a Cobas E601 C analyzer (Roche Diagnostics, Indianapolis, IN). Enzyme-linked immunosorbent assay (ELISA)was used for the detection of FGF23(EMD Millipore Corporation, Milford, MA, USA). Inorganic phosphorus (by Advia 1800, Siemens), and total calcium (by calorimetric method with Arsenazo, Advia 1800, Siemens) concentrations were measured in serum. Corrected calcium (mg/dL) was calculated as serum calcium (mg/dL) + 0.8 × [4 − serum albumin (g/dL)].

The complete osteocalcin (amino acid 1-49) is unstable in peripheral blood. The amino acid between 43-44 carboxyl end is easily hydrolyzed by protease, and the N-mid fragment is much more stable. In this study, the stable N-mid and intact osteocalcin in serum were detected by the kit (ECLIA Kit, Roche Diagnostics GmbH, Sandhofer strasse, Mannbeim, Germany). This result reflects the value of total osteocalcin in serum. It should be noted that this test does not distinguish between c-OC and unOC. A detailed test method can be found in the **Supplementary Materials**.

Abdominal aortic calcification (AAC) is assessed within 1 month of admission (11) by semiquantitative scoring of a plain lateral lumbar radiograph using previously validated 24-point aortic calcification scale (5). The scores, obtained separately for the anterior and posterill, result in a range from 0 to 6 for each vertebral level and 0 to 24 for the total AAC score (AACs). AACs was evaluated by one of the authors who was blinded to the other patient data.

### Statistical analysis

Data management and analysis were performed using SPSS Statistics 17.0 and a P-value of < 0.05 was considered statistically significant. All continuous variables that follow a normal distribution were expressed as mean ( ± standard deviation), and variables without normal distribution were expressed as median (interquartile range). Categorical variables were expressed as number (percentage). Student’s t test or Mann–Whitney U test (2-tailed) were performed to determine the between-group differences. The chi-squared test was used to compare categorical variables. Pearson correlation was used to determine the correlation, bivariate relationship, and strength of association among variables. A multiple logistics regression analysis was performed to investigate the determinants of AAC.

## Results

### The Characteristics of OC Level

The OC level of all 108 patients was 231.56 (25.92,361.33) ng/ml. It was higher than the normal range provided by our laboratory(**Table 1**).

**Table 1:** Comparison of OC levels and normal value median (5, 95 percentile) (ng/mL).

	Male			female	
	Patients results	Normal range		Patients results	Normal range
18-30(years)n=8	241(41-340)	40(24-70)	20-Before menopause n=14	245(26-388)	23(11-43)
30-50(years)n=20	243(30-390)	25(14-42)	After menopause n=28	238(27-371)	27(15-46)
50-70(years)n=35	211(29-319)	24(14-46)			
>70(years)n=3	231,225,203	27(13-48)			

### Comparison of Patients With Different AACs Levels

AACs scores higher than 5 were reported to have predictive value for cardiovascular disease in hemodialysis patients (12, 13). Based on this, all 108 MHD patients were divided into low AACs(AACs <5), and high AACs(AACs≥5) groups (13). The age, dialysis vintage, gender, DM and serum OC were significantly different among the groups, while there was no significant difference among the groups in BMI, KT/V, serum calcium, tAKP, iPTH and FGF23 (**Table 2**).

**Table 2:** Comparison of patients with different AACs levels.

	All patients	AACs<5 (n=56)	AACs ≥5 (n=52)	t/χ^2^/U	P value
Age(years)	55.12 ± 13.59	39.63 ± 13.21	58.71 ± 12.89	9.927^a^	0.000
Dialysis vintage(months)	44.4(24.3,96.7)	40.1(12.8,72.5)	45.4(15.3,98.7)	1.114^c^	0.038
Gender(male)	66(61.1%)	44(66.7%)	22(33.3%)	-5.627^b^	0.001
DM	15(13.9%)	2(13.3%)	13(86.6%)	2.163^b^	0.001
BMI	22.15 ± 3.46	21.64 ± 3.08	23.75 ± 3.25	0.342^a^	0.067
KT/V	1.52 ± 0.23	1.53 ± 0.27	1.54 ± 0.25	0.013^a^	0.873
calcium(mmol/l)	2.35 ± 0.25	2.30 ± 0.27	2.36 ± 0.21	0.353^a^	0.310
phosphorus (mmol/l)	1.60 ± 0.59	1.56 ± 0.71	1.64 ± 0.58	0.787^a^	0.601
tAKP(U/L)	73.30(53,123)	78.53(52,118)	72.33(60,103)	0.085^c^	0.754
iPTH(pg/ml)	502(179,739)	459(194,761)	634(215,807)	0.101^c^	0.476
FGF23(pg/ml)	5853.65(109,214370)	5203.42(117,193320)	103391(287,235108)	4.058^c^	0.062
OC(ng/ml)	231.56 (25.92,361.33)	217.06 (20.35,292.11)	256.37 (27.34,465.51)	6.522^c^	0.032

^a^Student’s t test, ^b^chi-squared test, ^c^Mann–Whitney U test.

AACs, Abdominal aortic calcification score; DM, diabetes mellitus; BMI, body mass index; KT/V, Urea Clearance Index; tAKP, total alkaline phosphatase; iPTH, intact parathyroid hormone; FGF23, fibroblast growth factor 23; OC, osteocalcin.

### OC Level and Correlation Analysis With Laboratory Indicators

Pearson correlation analysis revealed that OC was positively correlated with serum phosphorus (r = 0.511, P = 0.001), iPTH (r = 0.594, P = 0.0001), FGF23 (r = 0.485, P = 0.003), AACs (r = 0.413, P = 0.0201), and a negative correlation with age (r = –0.356, P = 0.039). There was no correlation of OC with serum calcium (r = 0.003, P = 0.977), total alkaline phosphatase (r = 0.171, P = 0.369), and dialysis vintage (r = 0.086, P = 0.565).

### Risk Factors for Vascular Calcification

Based on the level of AACs patients were divided into 2 groups, high AACs group(AACs≥5) and low AACs group(AACs <5). In the process of logistic regression, we grouped some variables. The assignment method of grouping variables was shown in **Table 3**, based mainly on published literature. Considering that age and DM, OC and PTH have a high degree of multicollinearity, when we removed DM and PTH from the model, R^2^ remained almost unchanged, we removed DM and PTH from the variables. Finally, the multiple logistics regression analysis revealed that age (OR = 1.14, P = 0.005) and OC (OR = 1.10, P = 0.008) are risk factors for high AACs (R^2^ = 0.412, P<0.0001) (**Table 3**).

**Table 3:** Multivariate logistic regression analysis for the presence of high AACs(AACs≥5).

Independent variable	OR(95%CI)	P-value
Age (per 10 years)	1.14(1.09 to 1.26)	0.005
Dialysis vintage (per 12 month)	1.08(0.90 to 2.27)	0.584
Gender male=1 female=2	0.88(0.73 to 1.64)	0.773
BMI^a^ (14) (kg/m^2^) <18.5 = 1 18.5-22.9 = 2 23.0-24.9 = 3 >=25 = 4	1.23(0.88to 1.51)	0.417
KT/V^a^ (15) <1.2 = 1 >=1.2 = 2	1.05(0.83 to 1.51)	0.718
calcium^a^ (10) (mmol/l) <2.1 = 1 2.1-2.5 = 2 >2.5 = 3	1.33(0.84 to 6.39)	0.612
phosphorus^a^ (10) (mmol/l) <1.45 = 1 1.45-1.78 = 2 >1.78 = 3	1.83(0.99 to 2.74)	0.393
tAKP^a^ (16) (U/L) <150 = 1 >150 = 2	1.13(0.78 to 1.56)	0.655
logFGF23	1.06(0.96 to 1.28)	0.594
OC^b^(ng/ml) <26 = 1 26-232 = 2 232-361 = 3 >361 = 4	1.10(1.01 to 1.18)	0.008

R^2^ = 0.412, P < 0.0001.

^a^grouping ranges based on values in references; b: quartile-based grouping.

OR, odds ratio; 95%CI, 95% confident interval; AACs, Abdominal aortic calcification score; BMI, body mass index; KT/V, Urea Clearance Index; tAKP, total alkaline phosphatase; FGF23, fibroblast growth factor 23; OC, osteocalcin.

## Discussion

MHD patients with CKD-MBD experience severe mixed intimal and medial vascular calcification, which contributes to the increased cardiovascular mortality and morbidity (2). VC is an active, complex process. At present, there is no good index to reflect the pathophysiological mechanism of vascular calcification, and guide clinical diagnosis and treatment. The classical markers, such as serum calcium (17), serum phosphorus (18), PTH (18), AKP (19) and FGF23 (20), are not specific and sensitive enough to reflect the pathophysiologic mechanisms underlying the process of VC (21). A review focuses on the pathophysiological mechanisms of vascular and valvular calcification in general population, list OC as one of the promoters in a table. In the body of this review, they emphasized that the circulating inactive, unOC reflect vitamin K deficiency and are markers of enhanced VC, especially in diabetic and CKD patients (22).

In vitro experiments, high OC level has been shown to stimulate VSMCs differentiation and mineralization in experimental model and OC has been found in calcified atherosclerotic plaque and calcified aortic valve (23). N-mid OC was positively associated with lower leg arterial calcification in advanced chronic kidney disease (24). In a recent study convincing evidences have been provided that OC may be implicated in the endothelial damage-related VC in patients with advanced chronic kidney disease *via* increasing the number of OC-positive endothelial progenitor cells (25). Giuseppe Cianciolo reported that VDR agonist therapy played a putative protective role to vascular calcification *via* decreased OC expression on circulating endothelial progenitor cells (26).

However, other studies in dialysis patients found no relationship between unOC and VC (27). Eiji Ishimura et al. found intact OC had no association with the presence of vascular calcification of the hand arteries of male hemodialysis patients (28). In contrast, studies in elderly men and hemodialysis patients showed that higher serum total OC levels were associated with lower AAC progression rate and lower 10-year all-cause mortality, suggesting a protective effect of OC in VC (29). Someone found an association between DM and decreased total OC and unOC levels in MHD patients, suggesting a potential protective role of OC in the bone, endocrine and vascular pathway (30).

Accurately, the level of OC in serum is influenced by many factors. Serum OC levels decreased with increasing age and increased with female sex in a general adult Danish population (31). PTH can promote osteoblasts to synthesize and secrete OC. PTH conjugates with the osteoblast PTH receptor on the cell membrane to regulate OC expression in order to increase bone formation, accelerate bone turnover, and increase the OC level in serum (32). FGF23 can upregulate OC lever *via* the JAK/STAT signaling pathway *in vitro* study (33). In patients with impaired renal function plasma OC levels are markedly elevated due to increased bone turnover and decreased renal elimination (34). Extracorporeal experiments also show that uremic serum itself promotes OC production (7).

As far as our study is concerned, OC elevated significantly in this group of MHD patients, which is consistent with previous research (35, 36). OC is positively correlated with phosphorus, iPTH, FGF23 and AACs, but the multiple logistics regression analysis revealed that only age and OC(OR=1.10, P=0.008)were risk factors for high AACs (≥5). There have been studies in dialysis patients showed increasing VC severity with age, regardless of factors such as dialysis vintage, dyslipidemia, PTH level and hypertension (21). It can be said that the effect of age on vascular calcification is well recognized. However, there are often conflicting conclusions about the relationship between other classical markers and vascular calcification (37, 38). This suggesting that the biochemical markers, vascular biomarkers and bone metabolism probably contribute to VC in a complex interconnected process. Pierre Delanaye et al. reported that a concordance between ΔPTH and Δbone biomarkers is observed in dialysis patients, but only after a long follow-up (39). For this reason, the revised 2017 KDIGO guidelines suggest that potential CKD-MBD therapies should be based on serial assessments of biomarkers, and thus on trends or variations (Δ), more than on one-single transversal result. Thus our study did not find a correlation between some classic indicators and VC, which may be related to the fact that this is only a cross-sectional study. Future research may have to focus on long-term follow-up study and multi interventional approach to prevent VC in CKD.

It’s important to note that total OC may not be a valuable measurement for risk of vascular calcification. It is reported that c-OC is the biologically active form. The hypothesis is that the functional vitamin K deficiency in hemodialysis patients with decreased c-OC is the trigger of calcification and not “unOC triggers calcification”. However, there has been difficulty in measuring ucOC and c-OC as few assays exist and it is unclear which assay system provides the most accurate measurements due to problems with comparability and heterogeneity of OC (40). Compared to healthy subjects, hemodialysis patients presented significantly reduced serum levels of active OC and increased serum levels of unOC. In a previous study which enrolled 189 hemodialysis patients together with 89 pre-dialysis CKD patients, the serum ucOC/intact OC ratio > 1.0 was observed in about 71.4% of hemodialysis patients especially those with high bone turnover (41). OC also displays a circadian rhythmicity with a nocturnal peak and thus timing of blood sampling may also contribute to variations in results (42). These factors all have an impact on the interpretation of the results.

It should be noted that serum OC levels and OC positive cells in the blood are completely different concepts and cannot be confused in the pathophysiological mechanisms of vascular calcification. Our study only discussed the correlation between serum OC levels and abdominal aortic calcification.

The current study is limited by the small number of patients, being single-center study. Vitamin K deficiency was not evaluated, The effect of external vitamin D supplementation was not assessed, either. The study was a cross-sectional observational studies, thus the cause–effect relationship cannot be concluded from the results.

In conclusion, our study shows that serum OC lever is associated with abnormal mineral parameters of CKD-MBD, OC was a risk factor for vascular calcification in this study, but this study did not classify osteocalcin as c-OC and unOC. Whether unOC is associated more directly with vascular calcification requires further study.

## Data Availability Statement

The original contributions presented in the study are included in the article/**Supplementary Material**. Further inquiries can be directed to the corresponding authors.

## Ethics Statement

The studies involving human participants were reviewed and approved by the ethics committee of 960th Hospital of the PLA Joint Logistics Support Force (protocol number: 20140214). The patients/participants provided their written informed consent to participate in this study.

## Author Contributions

All authors contributed to the article and approved the submitted version.

## Conflict of Interest

The authors declare that the research was conducted in the absence of any commercial or financial relationships that could be construed as a potential conflict of interest.
