# Supplementary material for: Osteocalcin and Abdominal Aortic Calcification in Hemodialysis Patients: An Observational Cross-Sectional Study
Source: Front Endocrinol (Lausanne). 2021 Mar 19;12:620350. doi: 10.3389/fendo.2021.620350 (PMC8018234; doi:10.3389/fendo.2021.620350)
Supplement: Supplementary file 1 [file Table_1.docx]

**Method:**

**Kid:Elecsys N-mid Osteocalcin**

Using Sandwich method, the whole process is completed in 18 minutes.

Step 1: The 20ul specimen, the biotinylated anti-N-mid osteocalcin monoclonal antibody, and the RU^a^ labeled anti-N-mid osteocalcin monoclonal antibody are mixed to form a sandwich complex.

Step 2: Add the Streptavidin coated particles, and the resulting complex is bonded to the particles by a reaction between the Biotin and Streptavidin.

- The reaction mixture is drawn to the measuring tank, the particles are adsorbed to the electrode by a magnet, and the unbonded material is washed away by the cleaning solution. A voltage applied to the electrode produces chemiluminescence, which is measured by Photomultiplier tubes.
- The test results are automatically checked from the standard curve by the machine.
- The curve is calibrated by a 2-point calibration of the instrument and is obtained by scanning the reagent bar code or electronic bar code into the original standard curve of the instrument.

2+

^a^ Tris(2,2’-bipyridyl)ruthenium(II)-complex (ru(bpy)

3
